# Plant Molecular Farming as a Strategy Against COVID-19 – The Italian Perspective

**DOI:** 10.3389/fpls.2020.609910

**Published:** 2020-12-14

**Authors:** Chiara Lico, Luca Santi, Selene Baschieri, Emanuela Noris, Carla Marusic, Marcello Donini, Emanuela Pedrazzini, Giovanni Maga, Rosella Franconi, Paola Di Bonito, Linda Avesani

**Affiliations:** ^1^Laboratory of Biotechnology, Biotechnologies and Agroindustry Division, Department of Sustainability, Italian National Agency for New Technologies, Energy and Sustainable Economic Development (ENEA), Rome, Italy; ^2^Department of Agriculture and Forest Science, Tuscia University, Viterbo, Italy; ^3^Institute for Sustainable Plant Protection, National Research Council IPSP-CNR, Turin, Italy; ^4^Institute for Sustainable Plant Protection, National Research Council IBBA-CNR, Turin, Italy; ^5^Institute of Molecular Genetics IGM-CNR “Luigi Luca Cavalli-Sforza,” Pavia, Italy; ^6^Laboratory of Biomedical Technologies, Health Technologies Division, Department of Sustainability, Italian National Agency for New Technologies, Energy and Sustainable Economic Development (ENEA), Rome, Italy; ^7^Department of Infectious Diseases, Viral Hepatitis, Oncoviruses and Retroviruses (EVOR) Unit, Istituto Superiore di Sanità, Rome, Italy; ^8^Department of Biotechnology, University of Verona, Verona, Italy

**Keywords:** therapeutics, vaccines, antibodies, diagnostics, recombinant proteins, transient expression, molecular farming, COVID-19

## Abstract

Severe acute respiratory syndrome coronavirus 2 (SARS-CoV-2) has killed more than 37,000 people in Italy and has caused widespread socioeconomic disruption. Urgent measures are needed to contain and control the virus, particularly diagnostic kits for detection and surveillance, therapeutics to reduce mortality among the severely affected, and vaccines to protect the remaining population. Here we discuss the potential role of plant molecular farming in the rapid and scalable supply of protein antigens as reagents and vaccine candidates, antibodies for virus detection and passive immunotherapy, other therapeutic proteins, and virus-like particles as novel vaccine platforms. We calculate the amount of infrastructure and production capacity needed to deal with predictable subsequent waves of COVID-19 in Italy by pooling expertise in plant molecular farming, epidemiology and the Italian health system. We calculate the investment required in molecular farming infrastructure that would enable us to capitalize on this technology, and provide a roadmap for the development of diagnostic reagents and biopharmaceuticals using molecular farming in plants to complement production methods based on the cultivation of microbes and mammalian cells.

## Introduction

Severe acute respiratory syndrome-related coronavirus 2 (SARS-CoV-2) is a new virus responsible for the COVID-19 pandemic, which is the worst public health crisis of this century^1^. The emergence of SARS-CoV-2 in late 2019 and its rapid spread in 2020 has posed several global challenges that demand new solutions in public healthcare and the biomedical research ecosystem (Webb et al., 2020). The former is facing acute pressure on hospital beds and frontline medical staff, whereas the latter is experiencing an explosion in new research, leading to a mountain of rapidly-disseminated literature often in the form of fast-tracked preprint articles. COVID-19 also dramatically highlighted the need for preparedness and long-term investments in platforms suitable for the rapid, flexible and sustainable production of medical countermeasures (diagnostics, vaccines, and therapeutics) against emerging, re-emerging, and bioterrorism-related infectious diseases (Franconi et al., 2018; Capell et al., 2020).

Italy was the first country in Europe to face a large-scale COVID-19 outbreak and it is one of the hardest-hit countries in the EU (Remuzzi and Remuzzi, 2020). Considering the situation with the resurgence of the disease after summer 2020, our national system must be prepared to react promptly by finding and exploiting new and flexible solutions. This requires an extraordinary effort aiming to capitalize on existing national expertise at different levels and will also provide assurance in the face of future pandemics. A major effort should also be done to build and potentiate the network with other countries in order to guide decisions in a global perspective always considering national peculiarities.

In this article, we discuss how plant molecular farming could provide practical solutions to address the outbreak of COVID-19 in Italy. Once effective vaccines and therapeutics are identified, it will be necessary to ramp up manufacturing to produce the massive numbers of doses needed to protect the entire population. The current global uneven distribution of manufacturing capacity is one of the current drawbacks of plant-based molecular farming as a realistic alternative to conventional expression systems (Capell et al., 2020; Tusé et al., 2020). Given this perspective, we have defined the product categories that would benefit most from molecular farming (diagnostics, vaccines, and therapeutics) and have calculated the quantities for each product category that would be needed to meet national demand, based on official Italian epidemiological reports in the February–June 2020 period.

Plant molecular farming encompasses a variety of different expression technologies, ranging from stable nuclear transformation (transgenic plants) or plastid transformation (transplastomic plants) to transient expression without stable transgene integration (Fischer and Buyel, 2020). In the latter case, this is achieved by the infiltration of adult wild-type plants—usually tobacco (*Nicotiana tabacum*) or its relative *Nicotiana benthamiana*—with strains of *Agrobacterium tumefaciens* or recombinant plant viral vectors carrying the appropriate transgene cassette (McDonald and Holtz, 2020). Given the urgent need for diagnostics, vaccines, and therapeutics for a rapidly-spreading novel or re-emerging disease, only transient expression systems provide the necessary speed and scalability, and we therefore focus on such systems in this article (Tusé et al., 2020).

## Diagnostic Reagents

The effective management of COVID-19 requires an increase in diagnostic capacity, particularly the development, manufacture, and stockpiling of assays to detect the SARS-CoV-2 genome and/or antigens itself or the antibodies it elicits. The former assays are used to confirm infections (thus ensuring effective quarantine measures and priority medical treatment) whereas antigenic tests or Rapid Tests, in point-of-care (PoC) format, have emerged as a valid approach in large screening of schools and vulnerable communities. On the other hand, antibody tests are used to assess prior infection and immunity status as the basis for epidemiological surveillance and vaccine studies. The number of different tests has increased rapidly and many are being marketed for point-of-care use. However, their accuracy has not been formally evaluated, and risks of bias, heterogeneity and limited generalizability have been reported for point-of-care testing (Bastos et al., 2020). In spite of this, frequent rapid tests are considered, in the moment of revising this paper, a “game change” tool before vaccines become available (Rubin, 2020).

Accurate antibody tests for COVID-19 require high-quality reagents, although differences between analytical and clinical sensitivity has not yet been defined for any test. The huge demand for diagnostic kits has highlighted not only the critical shortage of reagents (recombinant antigens and antibodies) but also the means to produce them.

Plants have already been shown to produce SARS-CoV antigens (Demurtas et al., 2016). The nucleoprotein (N), transiently expressed in *N. benthamiana*, was recognized by sera from Chinese SARS-convalescent patients around the time of the 2003 outbreak. Furthermore, the full-length membrane (M) protein was produced in plants but not in bacteria due to unanticipated toxicity (Carattoli et al., 2005). This provided proof of principle that plants could be used as a robust, rapid and flexible production system for SARS diagnostic reagents, potentially allowing the development of immunological assays for stockpiling in case of recurring SARS outbreaks (De Martinis et al., 2016).

Many other antigens have been produced in plants for diagnostic use or the preclinical/clinical evaluation of vaccine candidates, mostly by transient expression in *N. benthamiana*. The yields vary widely, as shown for a selection of antigens in Table 1. Antigens were selected among those that have been produced transiently in *N. benthamiana* plants and for which final purification yields were reported. In all these cases, the products were purified by affinity chromatography due to the presence of N-terminal or C-terminal affinity tags. Such tags are suitable for diagnostic reagents as long as they do not interfere with immunoreactivity, which is indeed the case for all the reported examples. The wide range of yields (1–220 μg/g fresh leaf mass, average 77.4 μg/g) shows that the feasibility of molecular farming for antigen manufacturing is exquisitely sensitive to the intrinsic nature of the product candidate (and also the quantification method). It is not yet possible to accurately predict yields based on a given candidate protein sequence, and empirical evaluation is therefore necessary, including the testing of multiple expression strategies – which is also facilitated by the scalability of transient expression systems (Gengenbach et al., 2020).

**TABLE 1:** Yields of selected purified molecules transiently expressed in plant systems.

**Diagnostic reagent**	**Yields (μg purified protein/g LFW)**	**References**
Bet V 1	23.4	Santoni et al., 2019
Dul51	4.9	Margolin et al., 2019
CAP256 SU	6.2	Margolin et al., 2019
NS1-ELP-ER	220	Marques et al., 2020
rHAO	200	Kanagarajan et al., 2012
SARS N protein	10	Demurtas et al., 2016
**Antibodies**		
mAb 2G12 (anti-HIV)	100	Sainsbury et al., 2010
mAb 6D8 (anti-Ebola)	500	Huang et al., 2010
mAb 2A10G6 (anti-Zika)	1,500	Diamos et al., 2020
mAb 4E10 (anti-HIV)	250	Zischewski et al., 2016
mAb M12 (tumor-specific)	2,000	Zischewski et al., 2016
Other therapeutics		
Griffithsin	1,000	O’Keefe et al., 2009
Griffithsin	519	Fuqua et al., 2015
**Subunit vaccine**		
HAC1	90	Shoji et al., 2011
HAI-05	50	Shoji et al., 2011
**VLP-vaccine**		
HBcAgD176	1,000	Peyret et al., 2015
HPV8 L1DC22	240	Matić et al., 2011
BPV1 LI	180	Love et al., 2012
HA Influenza Virus H5N1	50	D’Aoust et al., 2008
BTV	70	Thuenemann et al., 2013
HBcAg	2,380	Huang et al., 2006
CVPs-Flu epitope	1,100	Lico et al., 2009
CVPs-HIV epitope	600	Marusic et al., 2001

Most immunological diagnostic tests proposed for the detection of SARS-CoV-2 antibodies are based on the full-length viral spike (S) glycoprotein, the shorter external S1 segment, its receptor binding domain (RBD), or the N protein (Freeman et al., 2020; Klumpp-Thomas et al., 2020; Rosendal et al., 2020). For example, Amanat et al. (2020) reported a serological test based on the RBD in a classic direct ELISA design, in which the plate is coated with the recombinant RBD produced in mammalian or insect cells. Nevertheless, the surface-located S protein is under continuous selective pressure by the immune system, and new reagents would be needed whenever a new coronavirus subtype arises in the human population (Ou et al., 2020). In contrast, the N protein is highly conserved among coronaviruses and is abundantly expressed during the early stages of infection, triggering a strong antibody response. This makes it a suitable diagnostic reagent, in combination with other antigens such as RBD or the more conserved M protein, to develop pan-reactive coronavirus tests. The S and N proteins are typically produced in mammalian cells (HEK293) or insect cells infected with baculovirus vectors. The N protein has also been expressed in *Escherichia coli* (Carattoli et al., 2005; Pei et al., 2005), but differences between the bacterial and eukaryotic cytoplasmic compartments and the inability of bacteria to carry out eukaryotic-type post-translational modifications can reduce the affinity of such recombinant antigens for antibodies present in serum (Vankadari and Wilce, 2020).

The amount of coronavirus antigen required to detect IgG and IgM in patient serum ranged from 50–200 ng/well in a standard 96-well plate assay (Amanat et al., 2020; Freeman et al., 2020; Klumpp-Thomas et al., 2020; Rosendal et al., 2020). Based on this, we calculated the quantity of diagnostic reagent required to meet the demand in Italy, assuming a mid-range value for sensitive detection (100 ng/well), with two technical replicates for each individual and accounting for the fact that immunological tests would be required in quantities at least equivalent to the molecular assays currently used for COVID-19 diagnosis. This is because reliable serological tests are fundamental requirements for long-term follow-up studies in order to confirm the stability of (neutralizing) antibody responses and to define the threshold of neutralizing antibodies that prevents re-infection (Seow et al., 2020). In our calculation for the number of serological tests required in Italy, we considered the number of molecular tests performed in Italy between March and June 2020 as reported by the Italian Health Ministry^2^. Based on these criteria, and assuming average yields from Table 1 (77.4 μg/g), two technical repetitions for each patient, an average biomass of 10 g of infiltrated leaves per *N. benthamiana* plant during transient expression, and an average density of plants in a greenhouse of 75 plants per sq. m., we predict that approximately up to 6.5 kg of plant biomass would be required per month, corresponding to 8.7 sq. m. of greenhouse space, to produce enough reagents for all serological assays in Italy (Table 2).

**TABLE 2:** Estimates of the surface area of greenhouse necessary to produce the monthly amount of diagnostic or therapeutic reagents or vaccine molecules potentially required to fight SARS-CoV-2 in the March-June pandemic in Italy.

				**Therapeutics**					**Vaccine**				
	**Diagnostic Reagent^a^**				**Antibody^b^**		**Other^c^**			**Soluble Antigen^d^**		**VLP^e^**	
	**Number**	**Kg**		**Number of**	**Kg**		**Kg**		**Number**	**Kg**		**Kg**	
	**of tests**	**LFW**	**m^2^**	**persons treated**	**LFW**	**m^2^**	**LFW**	**m^2^**	**of doses**	**LFW**	**m^2^**	**LFW**	**m^2^**
Mar	483,623	1.25	1.67	10,376	153334	204445	40983	54644	3,6 × 10^7^	20696	27592	9388	12518
Apr	847,933	2.19	2.92	9,967	147297	196396	39370	52493					
May	2,523,838	6.52	8.69	2,756	40723	54298	10885	14513					
Jun	1,435,880	3.71	4.95	729	107775	14367	2880	3840					

*^*a*^Amount per test: 100 ng × 2*, *^*b*^9 g per dose*, *^*c*^3 g per dose*, *^*d*^40 μg per dose*, *^*e*^120 μg per dose*.

## Therapeutics

### Neutralizing Antibodies

Several trials suggest that passive immunotherapy based on the use of neutralizing antibodies from COVID-19 patients may be an important weapon in the fight against SARS-CoV-2 (Tortorici et al., 2020). Several pharmaceutical companies have already announced phase I trials of monoclonal neutralizing antibodies that protected animal models against SARS-CoV-2 (Ren et al., 2020). One of these studies is evaluating REGN-COV2, a cocktail of two antibodies that bind non-overlapping regions of the RBD (Baum et al., 2020; Hansen et al., 2020). Recently, Eli/Lilly disclosed the preliminary findings of randomized, double-blind, placebo-controlled Phase 2 study BLAZE-1 evaluating the combination of LY-CoV555 and LY-CoV016, two SARS-CoV-2 neutralizing antibodies, for the treatment of symptomatic COVID-19 in the outpatient setting^3^. The combination cohort enrolled recently diagnosed patients with mild-to-moderate COVID-19, who were assigned to 2,800 mg of each antibody or placebo. The combination therapy significantly reduced viral load at days 3, 7, and 11. Combination therapy has been generally well tolerated with no drug-related serious adverse events. These studies provide a first indication of an effective therapeutic dose of monoclonal antibodies against SARS-CoV-2 in the range of 5.6 g per patient.

Plants could provide an alternative system for the production of recombinant anti-COVID-19 antibodies given the efficient production of antibodies reported in plants, particularly by transient expression using viral vectors (Donini and Marusic, 2019). Recent examples of plant-derived neutralizing antibodies and their yields are listed in Table 1. The average yield of about 870 μg/g suggests that molecular farming could provide a useful complement for traditional manufacturing systems, especially when antibodies are required rapidly and on a large scale.

In a worst-case scenario, based on the Italian situation in the middle of March 2020, the proportion of COVID-19 patients admitted to an intensive care unit (ICU) was 9–11% (Remuzzi and Remuzzi, 2020). We calculated the amount of antibody required to meet the demand in Italy by assuming that all ICU patients would benefit from passive immunotherapy and that presumably the dose would be similar to that of the plant-derived cocktail ZMapp for the treatment of Ebola during the 2014 outbreak in West Africa – 9 g per patient (Mulangu et al., 2019). Based on these criteria, and again assuming average yields from Table 1 (870 μg/g), a final 70% recovery of highly purified mAb necessary for intra-venous administration route and the same values stated above for biomass and plant density in a greenhouse, we predict that approximately up to 153,000 kg of biomass would be required per month, corresponding to approximately 204,000 sq. m. of greenhouse space (Table 2).

### Other Proteins

In addition to passive and active immunization, antiviral proteins could provide an additional weapon against the virus during the infection phase, and other biologics could help to address the symptoms of COVID-19 such as the cytokine storm provoked by the initial wave of infection.

Among virus inhibitors, lectins are particularly interesting from the perspective of plant molecular farming because they are naturally produced in many higher plants and bind reversibly to carbohydrates. Given the frequent presence of glycans on the surface of viruses, lectins have been explored as antivirals and have been shown to block the infection cycle of HIV, cytomegalovirus, respiratory syncytial virus, influenza A, and also several coronaviruses. Indeed, more than 20 different plant lectins have already been shown to block infections by SARS-CoV, probably via selective binding to glycans on the S protein (Keyaerts et al., 2007). One of the best candidates is the algal lectin griffithsin (Table 1), which shows potent activity against SARS-CoV but limited toxicity toward human cells, and has been expressed at high levels in tobacco with average yields of 760 μg/g (O’Keefe et al., 2009; Fuqua et al., 2015; Hoelscher et al., 2018).

There is preliminary evidence that natural or artificial peptides can also inhibit viruses (Struck et al., 2011; Mookherjee et al., 2020) and short heterologous peptides have been successfully expressed in plants either independently or displayed on the surface of plant virus nanoparticles or virus-like particles (VLPs) by genetic fusion to the viral coat protein gene (Lico et al., 2012b). Furthermore, several approved drugs have been repurposed for COVID-19 including existing biologics used for anticoagulation therapy (Barrett et al., 2020) and immunomodulators used to block inflammatory cytokine signaling pathways (Rizk et al., 2020). Molecular farming could significantly reduce the manufacturing time and costs for such products, given that functional thrombin inhibitors and fibrinolytics (Abdoli Nasab et al., 2016; Pitek et al., 2018) as well as antibody-based immunomodulators (Jantan et al., 2015) have already been expressed successfully in plants.

Using the same strategy described above for passive immunotherapy, we considered the amount of griffithsin required in Italy for the treatment of 10% of COVID-19 patients admitted to ICUs, basing the dosing schedule on preclinical studies involving two intranasal doses of 5 mg/kg body mass daily for 4 days (O’Keefe et al., 2010). We came to a dose of 3 g, assuming an average patient mass of 74 kg (Pierlorenzi, 2010). Based on these criteria, and again assuming average yields from Table 1 (759.5 μg/g) and the same values stated above for biomass and plant density in a greenhouse, we predict that 41,000 kg of plant biomass would be required per month, corresponding to 55,000 sq. m. of greenhouse space (Table 2).

## Vaccines

The race to produce a COVID-19 vaccine began as soon as the SARS-CoV-2 genome sequence was published, involving hundreds of academic and industry research groups around the world using the most innovative biotechnology-based approaches. Thus far, 232 COVID-19 vaccine clinical trials have been registered and in some cases phase I safety data are already available^4^.

Conventional inactivated or live-attenuated vaccines against COVID-19 are likely to be efficacious but also difficult to manufacture and distribute widely, as well as presenting a risk of reverting to virulence. Subunit vaccines based on recombinant antigens, such as the S-protein/RBD (or VLPs displaying said antigens), are more attractive in terms of safety and manufacturing, and can also be produced by molecular farming in plants. Table 1 provides some examples of subunit and VLP vaccines produced in plants, and indicates average yields of 70 and 702.5 μg/g, respectively.

To determine the amount of vaccine antigen needed for a primary prevention approach in Italy (Table 2), we assumed that the dose of a soluble subunit vaccine would be the same as that recommended for the approved quadrivalent influenza vaccine, i.e., one shot of 40 μg of each antigen per individual, and that the dose of a VLP vaccine would be the same as that recommended for the VLP-based hepatitis B vaccine, i.e., one shot of 120 μg per individual (Mulangu et al., 2019). Such calculation is consistent with recent Phase 1 trials with the recombinant SARS-CoV-2 Spike protein adjuvated vaccine NVX-CoV2373, which showed promising immunogenic response and tolerability with an antigen dose of 50 μg, split in two administrations of 25 μg each 21 days apart (Keech et al., 2020). Also the ongoing Phase 2 clinical trial NCT04466085 with another vaccine based on recombinant RBD domain of the Spike protein, is testing doses ranging from 50 to 150 μg of antigen per individual. We also assumed that 60% of the Italian population (36,000,000) would need to be vaccinated in order to achieve herd immunity. Based on these criteria, and again assuming average yields from Table 1 (70 μg/g for soluble antigens, 703 μg/g for VLPs) and the same values stated above for biomass and plant density in a greenhouse, we calculate that approximately 21,000 kg of plant biomass and 28,000 sq. m. of greenhouse space would be required to satisfy the demand for a soluble antigen, whereas 9,400 kg of plant biomass and 12,500 sq. m. of greenhouse space would be required to produce sufficient quantities of the VLP vaccine (Table 2).

### Antigen-Based Vaccines

Neutralizing antibodies against coronaviruses often block interactions between the S protein and its receptor. In the case of SARS-CoV-2, this is angiotensin converting enzyme 2 (ACE2) primarily found on the surface of lung epithelial cells, although also in other tissues (Liu et al., 2020; Long et al., 2020). The S protein or parts thereof are therefore the favored as vaccine candidates, although the N and M proteins have also been considered. Previous coronavirus vaccines (against SARS-CoV and MERS-CoV) induced Th2-mediated immunopathology in animal models, and researchers are focusing on protein engineering strategies to minimize this effect (Koirala et al., 2020). One promising approach is to express the S1 or RBD parts of the S protein, focusing the immune response on the parts of the S protein that interact directly with ACE2 and thus increasing the likelihood of eliciting neutralizing antibodies. The S1/RBD surface is heavily glycosylated and these proteins are therefore more effective as antigens when expressed in eukaryotic cells (Walls et al., 2020). The highest yield of recombinant RBD has been achieved in yeast, reaching 400 mg/L in the fermentation supernatant at a production scale of up to 60 L (Chen et al., 2020). Another promising approach is the fusion of the S-protein or its components to the Fc region of a human IgG1 antibody, which prolongs its exposure to the immune system. An S-IgG1 fusion protein produced in CHO-K1 cells elicited promising immune responses in primates (Ren et al., 2020). According to the authors, following the theoretical vaccination schedule of the anti-VZV Shringrix vaccine (two doses of 50 μg) and considering a yield of 50 mg/L in CHO-K1 cells, 3 million doses of vaccine could be produced every 14 days in a 3000-L bioreactor. In the Italian scenario, with a herd immunity threshold of 60% (36,000,000 people), the required 72,000,000 doses could be produced in a single 3000-L bioreactor in approximately 1 year.

The S protein of several avian, swine and murine coronaviruses, as well as the N-terminal fragment of the SARS-CoV S protein, have been produced successfully in transgenic maize, potato, tomato, or tobacco plants by classic *Agrobacterium*-mediated transformation, or by display on the surface of plant viruses, and in all cases the products induced an immune response following oral delivery (Tuboly et al., 2000; Bae et al., 2003; Lamphear et al., 2004; Zhou et al., 2004) or nasal delivery (Koo et al., 1999). However, transient expression is more suitable for the speed and scale of production needed to address a rapidly-spreading disease live COVID-19. Two companies are known to be developing a plant-based subunit vaccine against COVID-19: Kentucky BioProcessing (Owensboro, KY, United States)^5^ and iBio (Bryan, TX, United States)^6^

### VLPs and Chimeric VLPs

VLPs are nanoparticles formed from virus structural proteins – they resemble the authentic virion but lack the virus genome. Chimeric VLPs include components of more than one virus, often because one virus provides the structural components of the VLP and another provides epitopes to display on the surface. VLP-based vaccines are already approved for immunization against hepatitis B virus, papillomaviruses, bluetongue virus, and Norwalk virus (Balke and Zeltins, 2019; Syomin and Ilyin, 2019) and more than 100 VLP-based candidate vaccines are currently undergoing clinical trials^7^. Several chimeric VLPs displaying coronavirus peptides identified by *in silico* analysis or docking are also in development (Kalitaa et al., 2020; Wang et al., 2020). VLPs based on plant viruses can be produced on a large scale by molecular farming (Lico et al., 2011, 2012b; Thuenemann et al., 2013). The examples listed in Table 1 range from simple VLPs based on a single viral protein to more complex structures containing up to four proteins (Marsian and Lomonossoff, 2016).

The strong potential of VLP vaccines made in plants is demonstrated by the VLPs developed by Medicago Inc. (QC, Canada) for vaccination against seasonal and pandemic influenza, which have reached phase III and II clinical trials, respectively. These vaccines are based on combinations of hemagglutinin proteins from different viral strains, which naturally assemble to form VLPs even in the absence of the other influenza virus structural proteins (D’Aoust et al., 2010). During the 2009 H1N1 pandemic, the company produced the first batch of research-grade vaccine only 3 weeks after receiving the A/H1N1 sequence (D’Aoust et al., 2010).

Although plant-derived SARS-CoV-2 VLPs have yet to be reported, the feasibility of this approach has been demonstrated by the successful production of other coronavirus VLPs in insect and mammalian cells (Lu et al., 2007, 2010; Bai et al., 2008; Lokugamage et al., 2008). This suggests that SARS-CoV2 VLPs could be assembled in plants by co-expressing the M, E, and S proteins. Medicago announced a program to develop a VLP-based COVID-19 vaccine candidate in July 2020, combining their recombinant coronavirus virus-like particle (CoVLP) technology with adjuvants from GlaxoSmithKline and Dynavax Technologies for the phase I trial^8^. A VLP-based COVID-19 vaccine program has also been announced by iBio Inc.^6^ This company was established with funding from the United States Defense Advanced Research Projects Agency (DARPA) and was part of the Blue Angel initiative to establish centers for the rapid delivery of medical countermeasures in response to emerging diseases, as demonstrated by the production of ∼10 million doses of influenza vaccine in only 1 month using its plant-based^9^.

In addition to SARS-CoV-2 VLPs, another potential route to a VLP-based COVID-19 vaccine is the production of chimeric VLPs displaying SARS-CoV-2 epitopes, as previously shown for human papillomavirus particles displaying influenza virus antigens (Matić et al., 2011) and hepatitis B virus particles displaying foot and mouth disease virus antigens (Huang et al., 2005). In the context of coronaviruses, this principle has been demonstrated by fusing the S1 domain of SARS-CoV to the transmembrane domain and C-terminus of avian influenzavirus hemagglutinin and expressing this with the avian influenzavirus matrix 1 protein in insect cells (Liu et al., 2011). Another successful strategy involves the RBD of the MERS-CoV S protein fused to the canine parvovirus structural protein VP2, resulting in VLPs that conferred protection and elicited neutralizing antibodies (Wang et al., 2017). Transient expression in plants should be able to produce similar VLPs on a larger scale, and more rapidly than any system based on cells cultivated in bioreactors. Plant viruses could also be used to display multiple immunogenic and immunomodulatory peptide epitopes (Lico et al., 2012a; Santoni et al., 2020), providing an effective strategy to induce simultaneous immune responses against different targets and to stimulate different components of the immune system – innate and adaptive, humoral and cell-mediated (Lico et al., 2013; Balke and Zeltins, 2019).

## Discussion

In many countries, schools have begun and workplaces reopened in order to restart the economy after the devastating effects of the COVID-19 lockdown, while several countries are already experiencing a second epidemic wave (Ali, 2020). In this context, we have shown how plant molecular farming could contribute to an effective response strategy in a country like Italy, where expertise in this field is mainly restricted to the research sector. National experts were gathered to envisage the scenario of a second epidemic wave, with a distribution and infection rate similar to the primary wave, in order to quantify the potential of plant molecular farming as a manufacturing platform for the production of diagnostic reagents, therapeutics and vaccines.

In all our forecasts, we restricted the platform technology to transient expression, because this allows the rapid initiation and scale-up of production, generating the first batches in only a few weeks (Hefferon, 2014). Furthermore, the large-scale production of recombinant proteins by transient expression is already considered consistent with good manufacturing practice in several countries, which is a prerequisite for the manufacture of pharmaceutical products (Fischer et al., 2012). Plant molecular farming would require only minimal investment compared to the expansion of fermenter infrastructure for microbes or animal cells, and would be much more flexible in the face of emergency scenarios as seen with COVID-19.

Recent commercial investments in large-scale automated vertical farming facilities for the production of biopharmaceuticals have shown that it is possible to process up to 7.58 kg of biomass per month per sq. m. (Holtz et al., 2015) and more recently Buyel et al. (2017) reported for a vertical farming unit yields of 68.25 kg of biomass per month per sq. m. Therefore, two such facilities would be able to process enough biomass in one week to meet Italy’s entire demand for a VLP-based vaccine (sufficient to achieve herd immunity) and for diagnostic reagents (sufficient to test the entire population) with only 10% of the capital costs required for fermenter-based infrastructure. For the production of neutralizing antibodies and antivirals such as griffithsin, our projection suggests that plant molecular farming could provide additional capacity, thus complementing other biopharmaceutical production platforms to ramp up the speed and scale of manufacturing at the time of greatest need. The footprint of such production facilities could also be reduced by advances that improve the yield of recombinant protein in plant tissues (Barbante et al., 2008; Rigano et al., 2009; Avesani et al., 2010) thus lowering unit costs (Nandi et al., 2016). Our projections for Italy also suggest that investments in molecular farming infrastructure could provide a valuable approach for any country looking to improve its preparedness for a second wave of COVID-19 and future epidemic or pandemic diseases. Furthermore, this technology has the main advantage of easy-scalability that enables to set rapidly the platform on the basis of the ongoing needs; in a rapidly changing scenario, such as the one that we are experiencing, this aspect may help in directing national decisions also on the basis of global experiences, taking advantage from the global network that has been recently established.

In this regard, to help consolidate molecular farming in Europe, the network could benefit from the service offered by the EU Research Infrastructures (RIs) that are being established in Europe according to the ESFRI roadmap (program H2020-EU.1.4.1.2) and will be fully operational in the coming years. RIs are facilities, resources or services, identified by European research communities to conduct and to support top-level research activities in their domains. In particular, IBISBA (Industrial Biotechnology Innovation and Synthetic Biology Accelerator^10^) is an Engineering Biology Research Infrastructure that brings together research organizations providing experimental and *in silico* services for industry and academia, to accelerate bio-based manufacturing processes.

Furthermore, we believe that joint investments from public and private funding may help to reach this ambitious goal, having a major impact on the complex health national eco-system and possibly contributing in a global perspective to adopt effective countermeasures in emergency situations as the current one.

## Data Availability Statement

The original contributions presented in the study are included in the article/supplementary material, further inquiries can be directed to the corresponding author.

## Author Contributions

LA coordinated the single parts and assembled the final version. All authors wrote the article.

## Conflict of Interest

The authors declare that the research was conducted in the absence of any commercial or financial relationships that could be construed as a potential conflict of interest.

## Footnotes

1 https://coronavirus.jhu.edu/

2 http://opendatadpc.maps.arcgis.com/apps/opsdashboard/index.html#/b0c68bce2cce478eaac82fe38d4138b1

3 https://clinicaltrials.gov/ct2/show/NCT04427501

4 https://clinicaltrials.gov/ (search term = vaccine and COVID-19)

5 https://www.bat.com/group/sites/UK__9D9KCY.nsf/vwPagesWebLive/DOBN8QNL

6 https://www.ibioinc.com/therapeutics-and-vaccines/ibio-201

7 https://clinicaltrials.gov/

8 https://www.medicago.com/en/newsroom/medicago-begins-phase-i-clinical-trials-for-its-covid-19-vaccine-candidate/

9 https://www.globenewswire.com/Tracker?data=_2Yc5Uv_N4cIurClgphsjzVbq4NvRaSxtXk1OPx9fG_oeiLQR7BDxDPNyC1SS5rtmVNyZIhMkgfgshOzS1F8nQ_-XWg65SAx7NHQATzShrRT2s0NubFL7PbzaOYg-QR6

10 http://www.ibisba.eu

## References

[B2] Abdoli Nasab M. Jalali Javaran M. Cusido R. M. Palazon J. (2016). Purification of recombinant tissue plasminogen activator (rtPA) protein from transplastomic tobacco plants. *Plant Physiol. Biochem.* 108 139–144. 10.1016/j.plaphy.2016.06.029 27428368

[B3] Ali I. (2020). COVID-19: are we ready for the second wave? *Disaster Med. Public Health Prep.* 7 1–3. 10.1017/dmp.2020.149 PMC7239772 32379015

[B4] Amanat F. Stadlbauer D. Strohmeier S. Nguyen T. H. O. Chromikova V. McMahon M. (2020). A serological assay to detect SARS-CoV-2 seroconversion in humans. *Nat. Med.* 26 1033–1036. 10.1038/s41591-020-0913-5 32398876 PMC8183627

[B5] Avesani L. Vitale A. Pedrazzini E. Devirgilio M. Pompa A. Barbante A. (2010). Recombinant human GAD65 accumulates to high levels in transgenic tobacco plants when expressed as an enzymatically inactive mutant. *Plant Biotech. J.* 8 1–11. 10.1111/j.1467-7652.2010.00514.x 20374524

[B6] Bae J.-L. Lee J.-G. Kang T.-J. Jang H.-S. Jang Y.-S. Yang M.-S. (2003). Induction of antigen-specific systemic and mucosal immune responses by feeding animals transgenic plants expressing the antigen. *Vaccine* 21 4052–4058. 10.1016/s0264-410x(03)00360-8 12922142

[B7] Bai B. Hu Q. Hu H. Zhou P. Shi Z. Meng J. (2008). Virus-like particles of SARS-like coronavirus formed by membrane proteins from different origins demonstrate stimulating activity in human dendritic cells. *PLoS One* 3:e2685. 10.1371/journal.pone.0002685 18628832 PMC2441860

[B8] Balke I. Zeltins A. (2019). Use of plant viruses and virus-like particles for the creation of novel vaccines. *Adv. Drug Deliv. Rev.* 145 119–129. 10.1016/j.addr.2018.08.007 30172923

[B9] Barbante A. Irons S. Hawes C. Frigerio L. Vitale A. Pedrazzini E. (2008). Anchorage to the cytosolic face of the ER membrane: a new strategy to stabilize a cytosolic recombinant antigen in plants. *Plant Biotech. J.* 6 560–575. 10.1111/j.1467-7652.2008.00342.x 18444969

[B10] Barrett C. D. Moore H. B. Moore E. E. McIntyre R. C. Moore P. K. Burke J. (2020). Fibrinolytic therapy for refractory COVID-19 acute respiratory distress syndrome: scientific rationale and review. *Res. Pract. Thromb Haemost.* 4 524–531. 10.1002/rth2.12357 32542213 PMC7267116

[B11] Bastos L. M. Tavaziva G. Abidi S. K. Campbell J. R. Haraoui L. P. Johnston J. C. (2020). Diagnostic accuracy of serological tests for COVID-19: systematic review and meta-analysis. *BMJ* 370:m2516. 10.1136/bmj.m2516 32611558 PMC7327913

[B12] Baum A. Fulton B. O. Wloga E. Copin R. Pascal K. E. Russo V. (2020). Antibody cocktail to SARS-CoV-2 spike protein prevents rapid mutational escape seen with individual antibodies. *Science* 369:eabd0831. 10.1126/science.abd0831 32540904 PMC7299283

[B13] Buyel J. F. Twyman R. M. Fischer R. (2017). Very large-scale production of antibodies in plants: the bioligization of manufacturing. *Biotechnol. Adv.* 35 458–465. 10.1016/j.biotechadv.2017.03.011 28347720

[B14] Capell T. Twyman R. M. Armario-Najera V. Ma J. K.-C. Schillberg S. Christou P. (2020). Potential applications of plant biotechnology against SARS-CoV-2. *Trends Plant Sci.* 25 635–643. 10.1016/j.tplants.2020.04.009 32371057 PMC7181989

[B15] Carattoli A. Di Bonito P. Grasso F. Giorgi C. Blasi F. Niedrig M. (2005). Recombinant protein-based ELISA and immuno-cytochemical assay for the diagnosis of SARS. *J. Med. Virol.* 76 137–142. 10.1002/jmv.20338 15834868 PMC7166672

[B16] Chen W.-H. Hotez P. J. Bottazzi M. E. (2020). Potential for developing a SARS-CoV receptor-binding domain (RBD) recombinant protein as a heterologous human vaccine against coronavirus infectious disease (COVID)-19. *Hum. Vaccines Immunother.* 16 1239–1242. 10.1080/21645515.2020.1740560 32298218 PMC7482854

[B17] D’Aoust M.-A. Couture M. M.-J. Charland N. Trépanier S. Landry N. Ors F. (2010). The production of hemagglutinin-based virus-like particles in plants: a rapid, efficient and safe response to pandemic influenza. *Plant Biotechnol. J.* 8 607–619. 10.1111/j.1467-7652.2009.00496.x 20199612

[B18] D’Aoust M. A. Lavoie P. O. Couture M. M. Trepanier S. Guay J. M. Dargis M. (2008). Influenza virus−like particles produced by transient expression in Nicotiana benthamiana induce a protective immune response against a lethal viral challenge in mice. *Plant Biotechnol. J.* 6 930–940. 10.1111/j.1467-7652.2008.00384.x 19076615

[B19] De Martinis D. Rybicki E. P. Fujiyama K. Franconi R. Benvenuto E. (2016). Molecular farming: fast, scalable, cheap, sustainable. *Front. Plant Sci.* 7:1148. 10.3389/fpls.2016.01148 27536308 PMC4971507

[B20] Demurtas O. C. Massa S. Illiano E. De Martinis D. Chan P. K. S. Di Bonito P. (2016). Antigen production in plant to tackle infectious diseases flare up: the case of SARS. *Front. Plant Sci.* 7:54. 10.3389/fpls.2016.00054 26904039 PMC4742786

[B21] Diamos A. G. Hunter J. G. L. Pardhe M. D. Rosenthal S. H. Sun H. Foster B. C. (2020). High level production of monoclonal antibodies using an optimized plant expression system. *Front. Bioeng. Biotechnol.* 17:472. 10.3389/fbioe.2019.00472 32010680 PMC6978629

[B22] Donini M. Marusic C. (2019). Current state-of-the-art in plant-based antibody production systems. *Biotechnol. Lett.* 41 335–346. 10.1007/s10529-019-02651-z 30684155

[B23] Fischer R. Buyel J. F. (2020). Molecular farming–the slope of enlightenment. *Biotechnol. Adv.* 40:107519. 10.1016/j.biotechadv.2020.107519 31954848

[B24] Fischer R. Schillberg S. Hellwig S. Twyman R. M. Drossard J. (2012). GMP issues for recombinant plant-derived pharmaceutical proteins. *Biotechnol. Adv.* 30 434–439. 10.1016/j.biotechadv.2011.08.007 21856403

[B25] Franconi R. Illiano E. Paolini F. Massa S. Venuti A. Demurtas O. C. (2018). “Rapid and low-cost tools derived from plants to face emerging/re-emerging infectious diseases and bioterrorism agents,” in *Defence against Bioterrorism. NATO Science for Peace and Security Series A: Chemistry and Biology*, eds Radosavljevic V. Banjari I. Belojevic G. (Dordrecht: Springer). 10.1007/978-94-024-1263-5_10

[B26] Freeman B. Lester S. Mils L. Moye M. Abiona O. Hutchinson G. B. (2020). Validation of a SARS-CoV-2 spike protein ELISA for use in contact investigations and sero-surveillance. *bioRxiv* [Preprint]. 10.1101/2020.04.24.057323 32511332 PMC7239067

[B27] Fuqua J. L. Wanga V. Palmer K. E. (2015). Improving the large scale purification of the HIV microbicide, griffithsin. *BMC Biotechnol.* 15:12. 10.1186/s12896-015-0120-5 25887919 PMC4349730

[B28] Gengenbach B. B. Opdensteinen P. Buyel J. F. (2020). Robot cookies–plant cell packs as an automated high-throughput screening platform based on transient expression. *Front. Bioeng. Biotechnol.* 8:393. 10.3389/fbioe.2020.00393 PMC7214789 32432097

[B29] Hansen J. Baum A. Pascal K. E. Russo V. Giordano S. Wloga E. (2020). Studies in humanized mice and convalescent humans yield a SARS-CoV-2 antibody cocktail. *Science* 369:eabd0827. 10.1126/science.abd0827 32540901 PMC7299284

[B30] Hefferon K. (2014). Plant virus expression vector development: new perspectives. *Biomed. Res. Int.* 2014:785382. 10.1155/2014/785382 24745025 PMC3972958

[B31] Hoelscher M. Tiller N. Teh A. Y. Wu G. Z. Ma J. K. Bock R. (2018). High-level expression of the HIV entry inhibitor griffithsin from the plastid genome and retention of biological activity in dried tobacco leaves. *Plant Mol. Biol.* 97 357–370. 10.1007/s11103-018-0744-7 29948657 PMC6061503

[B32] Holtz B. R. Berquist B. R. Bennett L. D. Kommineni V. J. M. Munigunti R. K. White E. L. (2015). Commercial-scale biotherapeutics manufacturing facility for plant-made pharmaceuticals. *Plant Biotechnol. J.* 13 1180–1190. 10.1111/pbi.12469 26387511

[B33] Huang Y. Liang W. Wang Y. Zhou Z. Pan A. Yang X. (2005). Immunogenicity of the epitope of the foot-and-mouth disease virus fused with a hepatitis B core protein as expressed in transgenic tobacco. *Viral Immunol.* 18 668–677. 10.1089/vim.2005.18.668 16359233

[B34] Huang Z. Phoolcharoen W. Lai H. Piensook K. Cardineau G. Zeitlin L. (2010). High-level rapid production of full-size monoclonal antibodies in plants by a single-vector DNA replicon system. *Biotechnol. Bioeng.* 106 9–17. 10.1002/bit.22652 20047189 PMC2905544

[B35] Huang Z. Santi L. LePore K. Kilbourne J. Arntzen C. J. Mason H. S. (2006). Rapid, high-level production of hepatitis B core antigen in plant leaf and its immunogenicity in mice. *Vaccine* 24 2506–2513. 10.1016/j.vaccine.2005.12.024 16417953

[B36] Jantan I. Ahmad W. Bukhari S. N. A. (2015). Plant-derived immunomodulators: an insight on their preclinical evaluation and clinical trials. *Front. Plant Sci.* 6:655. 10.3389/fpls.2015.00655 26379683 PMC4548092

[B37] Kalitaa P. Padhic A. K. Zhang K. Y. J. Tripathi T. (2020). Design of a peptide-based subunit vaccine against novel coronavirus SARS-CoV-2. *Microbial Pathogen.* 145:104236 10.1016/j.micpath.2020.104236 PMC7196559 32376359

[B38] Kanagarajan S. Tolf C. Lundgren A. Waldenström J. Brodelius P. E. (2012). Transient expression of hemagglutinin antigen from low pathogenic avian influenza A (H7N7) in nicotiana benthamiana. *PLoS One* 7:e33010. 10.1371/journal.pone.0033010 22442675 PMC3307706

[B39] Keech C. Albert G. Cho I. Robertson A. Reed P. Neal S. (2020). Phase 1-2 trial of a SARS-CoV-2 recombinant spike protein nanoparticle vaccine. *N. Engl. J. Med.* 2020:NEJMoa2026920. 10.1056/NEJMoa2026920.) 32877576 PMC7494251

[B40] Keyaerts E. Vijgen L. Pannecouque C. Van Damme E. Peumans W. Egberink H. (2007). Plant lectins are potent inhibitors of coronaviruses by interfering with two targets in the viral replication cycle. *Antiviral Res.* 75 179–187. 10.1016/j.antiviral.2007.03.003 17428553 PMC7114093

[B41] Klumpp-Thomas C. Drew M. Husberger S. Snead K. Fay M. P. Mehalko J. (2020). Standardization of enzyme-linked immunosorbent assays for serosurveys of the SARS-CoV-2 pandemic using clinical and at-home blood sampling. *medRxiv* [Preprint]. 10.1101/2020.05.21.20109280 33397956 PMC7782755

[B42] Koirala A. Joo Y. J. Khatami A. Chiu C. Britton P. N. (2020). Vaccines for COVID-19: the current state of play. *Paediatric Respir. Rev.* 35 43–49. 10.1016/j.prrv.2020.06.010 32653463 PMC7301825

[B43] Koo M. Bendahmane M. Lettieri G. A. Paoletti A. D. Lane T. E. Fitchen J. H. (1999). Protective immunity against murine hepatitis virus (MHV) induced by intranasal or subcutaneous administration of hybrids of tobacco mosaic virus that carries an MHV epitope. *Proc. Natl. Acad. Sci. U.S.A.* 96 7774–7779. 10.1073/pnas.96.14.7774 10393897 PMC22137

[B44] Lamphear B. J. Jilka J. M. Kesl L. Welter M. Howard J. A. Streatfield S. J. (2004). A corn-based delivery system for animal vaccines: an oral transmissible gastroenteritis virus vaccine boosts lactogenic immunity in swine. *Vaccine* 22 2420–2424. 10.1016/j.vaccine.2003.11.066 15193404 PMC7126512

[B45] Lico C. Buriani G. Capuano F. Benvenuto E. Baschieri S. (2011). *Influenza Vaccines: New Perspectives from Plants. Plant-derived Vaccines: Technologies & Applications.* London: Future Science Group, 104–115. 10.2217/EBO.11.73

[B46] Lico C. Mancini C. Italiani P. Betti C. Boraschi D. Benvenuto E. (2009). Plant-produced potato virus X chimeric particles displaying an influenza virus-derived peptide activate specific CD8+ T cells in mice. *Vaccine* 27 5069–5076. 10.1016/j.vaccine.2009.06.045 19563889

[B47] Lico C. Marusic C. Capuano F. Buriani G. Benvenuto E. Baschieri S. (2012a). *Plant-based Vaccine Delivery Strategies Innovation in Vaccinology: from Design, through to Delivery and Testing.* Dordrecht: Springer Science+Business Media.

[B48] Lico C. Santi L. Twyman R. M. Pezzotti M. Avesani L. (2012b). The use of plants for the production of therapeutic human peptides. *Plant Cell Rep.* 31 439–451. 10.1007/s00299-011-1215-7 22218674

[B49] Lico C. Schoubben A. Baschieri S. Blasi P. Santi L. (2013). Nanoparticles in biomedicine: new insights from plant viruses. *Curr. Med. Chem.* 20 3471–3487. 10.2174/09298673113209990035 23745557

[B50] Liu A. Li Y. Peng J. Huang Y. Xu D. (2020). Antibody responses against SARS-CoV-2 in COVID-19 patients. *J. Med. Virol.* 30 1–5. 10.1002/jmv.26241 32603501 PMC7362084

[B51] Liu Y. V. Massare M. J. Barnard D. L. Kort T. Nathan M. Wang L. (2011). Chimeric severe acute respiratory syndrome coronavirus (SARS-CoV) S glycoprotein and influenza matrix 1 efficiently form virus-like particles (VLPs) that protect mice against challenge with SARS-CoV. *Vaccine* 29 6606–6613. 10.1016/j.vaccine.2011.06.111 21762752 PMC3165014

[B52] Lokugamage K. G. Yoshikawa-Iwata N. Ito N. Watts D. M. Wyde P. R. Wang N. (2008). Chimeric coronavirus-like particles carrying severe acute respiratory syndrome coronavirus (SCoV) S protein protect mice against challenge with SCoV. *Vaccine* 26 797–808. 10.1016/j.vaccine.2007.11.092 18191004 PMC2267761

[B53] Long Q. X. Liu B. Z. Deng H. J. Wu G. C. Deng K. Chen Y. K. (2020). Antibody responses to SARS-CoV-2 in patients with COVID-19. *Nat. Med.* 26 845–848. 10.1093/cid/ciaa344 32350462

[B54] Love A. J. Chapman S. N. Matic S. Noris E. Lomonossoff G. Taliansky M. (2012). In planta production of a candidate vaccine against bovine papillomavirus type 1. *Planta* 236 1305–1313. 10.1007/s00425-012-1692-0 22718313

[B55] Lu B. Huang Y. Huang L. Li B. Zheng Z. Chen Z. (2010). Effect of mucosal and systemic immunization with virus-like particles of severe acute respiratory syndrome coronavirus in mice. *Immunology* 130 254–261. 10.1111/j.1365-2567.2010.03231.x 20406307 PMC2878469

[B56] Lu X. Chen Y. Bai B. Hu H. Tao L. Yang J. (2007). Immune responses against severe acute respiratory syndrome coronavirus induced by virus-like particles in mice. *Immunology* 122 496–502. 10.1111/j.1365-2567.2007.02676.x 17680799 PMC2266036

[B57] Margolin E. Chapman R. Meyers A. E. van Diepen M. T. Ximba P. Hermanus T. (2019). Production and immunogenicity of soluble plant-produced HIV-1 subtype C envelope gp140 immunogens. *Front. Plant Sci.* 10:1378. 10.3389/fpls.2019.01378 31737007 PMC6831737

[B58] Marques L. E. C. Silva B. B. Fireman Dutra R. Tramontina Florean E. O. P. Menassa R. Guedes M. I. F. (2020). Transient expression of dengue virus NS1 antigen in *Nicotiana benthamiana* for use as a diagnostic antigen. *Front. Plant Sci.* 16:1674. 10.3389/fpls.2019.01674 32010161 PMC6976532

[B59] Marsian J. Lomonossoff G. P. (2016). Molecular pharming-VLPs made in plants. *Curr. Opin. Biotech.* 37 201–206. 10.1016/j.copbio.2015.12.007 26773389

[B60] Marusic C. Rizza P. Lattanzi L. Mancini C. Spada M. Belardelli F. (2001). Chimeric plant virus particles as immunogens for inducing murine and human immune responses against human immunodeficiency virus type 1. *J. Virol.* 75 8434–8439. 10.1128/JVI.75.18.8434-8439.2001 11507188 PMC115088

[B61] Matić S. Rinaldi R. Masenga V. Noris E. (2011). Efficient production of chimeric Human papillomavirus 16 L1 protein bearing the M2e influenza epitope in *Nicotiana benthamiana* plants. *BMC Biotechnol.* 11:106. 10.1186/1472-6750-11-106 22085463 PMC3248878

[B62] McDonald K. A. Holtz R. B. (2020). From farm to finger prick—a perspective on how plants can help in the fight against COVID-19 front. *Bioeng. Biotechnol.* 8:782. 10.3389/fbioe.2020.00782 32714921 PMC7351482

[B63] Mookherjee N. Anderson M. A. Haagsman H. P. Davidson D. J. (2020). Antimicrobial host defence peptides: functions and clinical potential. *Nat. Rev. Drug Discovery* 19 311–332. 10.1038/s41573-019-0058-8 32107480

[B64] Mulangu S. Dodd L. E. Davey R. T. Jr. Mbaya O. T. Proschan M. Mukadi D. (2019). A randomized, controlled trial of ebola virus disease therapeutics. *N. Engl. J. Med.* 381 2293–2303. 10.1056/NEJMoa1910993 31774950 PMC10680050

[B65] Nandi S. Kwong A. T. Holtz B. R. Erwin B. R. Marcel S. McDonald K. A. (2016). Techno-economic analysis of a transient plant-based platform for monoclonal antibody production. *MABS* 8 1456–1466. 10.1080/19420862.2016.1227901 27559626 PMC5098453

[B66] O’Keefe B. R. Giomarelli B. Bernard D. L. Shenoy S. R. Chan P. K. S. McMahon J. B. (2010). Broad-spectrum in vitro activity and in vivo efficacy of the antiviral protein griffithsin against emerging viruses of the family coronaviridae. *J. Virol.* 84 2511–2521. 10.1128/JVI.02322-09 20032190 PMC2820936

[B67] O’Keefe B. R. Vojdani F. Buffa V. Shattock R. J. Montefiori D. C. Bakke J. (2009). Scalable manufacture of HIV-1 entry inhibitor griffithsin and validation of its safety and efficacy as a topical microbicide component. *Proc. Natl. Acad. Sci. U.S.A.* 106 6099–6104. 10.1073/pnas.0901506106 19332801 PMC2662964

[B68] Ou X. Liu Y. Lei X. Li P. Mi D. Ren L. (2020). Characterization of spike glycoprotein of SARS-CoV-2 on virus entry and its immune cross-reactivity with SARS-CoV. *Nat. Commun.* 11:1620. 10.1038/s41467-020-15562-9 32221306 PMC7100515

[B69] Pei H. Liu J. Sun C. Wang C. Lu Y. Ding J. (2005). Expression of SARS-coronavirus nucleocapsid protein in *Escherichia coli* and *Lactococcus lactis* for serodiagnosis and mucosal vaccination. *Appl. Microbiol. Biotechnol.* 68 220–227. 10.1007/s00253-004-1869-y 15660214 PMC7079895

[B70] Peyret H. Gehin A. Thuenemann E. C. Blond D. El Turabi A. Beales L. (2015). Tandem fusion of hepatitis B core antigen allows assembly of virus-like particles in bacteria and plants with enhanced capacity to accommodate foreign proteins. *PLoS One* 10:e0120751. 10.1371/journal.pone.0120751 25830365 PMC4382129

[B71] Pierlorenzi G. (2010). *L’italia si Misura.* Rome: Aracne Editrice.

[B72] Pitek A. S. Park J. Wang Y. Gao H. Hu H. Simon D. I. (2018). Delivery of thrombolytic therapy using rod-shaped plant viral nanoparticles decreases the risk of hemorrhage. *Nanoscale* 10 16547–16555. 10.1039/c8nr02861c 30137088 PMC6145846

[B73] Remuzzi A. Remuzzi G. (2020). COVID-19 and Italy: what next? *Lancet* 395 1225–1228. 10.1016/s0140-6736(20)30627-9 32178769 PMC7102589

[B74] Ren W. Sun H. Gao G. Chen J. Sun S. Zhao R. (2020). Recombinant SARS-CoV-2 spike S1-Fc fusion protein induced high levels of neutralizing responses in nonhuman primates. *Vaccine* 38 5653–5658. 10.1016/j.vaccine.2020.06.066 32651113 PMC7311893

[B75] Rigano M. M. Manna C. Giulini A. Pedrazzini E. Capobianchi M. Castilletti C. (2009). Transgenic chloroplasts are efficient sites for high-yield production of the vaccinia virus envelope protein A27L in plant cells. *Plant Biotech. J.* 6 577–591. 10.1111/j.1467-7652.2009.00425.x 19508274

[B76] Rizk J. G. Kalantar-Zadeh K. Mehra M. R. Lavie C. J. Rizk Y. Forthal D. N. (2020). Pharmaco-immunomodulatory therapy in COVID-19. *Drugs* 80 1267–1292. 10.1007/s40265-020-01367-z 32696108 PMC7372203

[B77] Rosendal E. Wigren J. Groening R. Yongdae K. Nilsson E. Sharma A. (2020). Detection of asymptomatic SARS-CoV-2 exposed individuals by a sensitive S-based ELISA. *medRxiv* [Preprint]. 10.1101/2020.06.02.20120477 32577692 PMC7302301

[B78] Rubin R. (2020). The challenges of expanding rapid tests to curb COVID-19. *JAMA* 324 1813–1815. 10.1001/jama.2020.21106 33084882

[B79] Sainsbury F. Sack M. Stadlmann J. Quendler H. Fischer R. Lomonossoff G. P. (2010). Rapid transient production in plants by replicating and non-replicating vectors yields high quality functional anti-HIV antibody. *PLoS One* 5:e13976. 10.1371/journal.pone.0013976 21103044 PMC2980466

[B80] Santoni M. Ciardiello M. A. Zampieri R. Pezzotti M. Giangrieco L. Rafaiani C. (2019). Plant-made Bet V1 for molecular diagnosis. *Front. Plant Sci.* 10:1273. 10.3389/fpls.2019.01273 31649716 PMC6795700

[B81] Santoni M. Zampieri R. Avesani L. (2020). Plant virus nanoparticles for vaccine applications. *Curr. Protein Pept. Sci.* 21 344–356. 10.2174/1389203721666200212100255 32048964

[B82] Seow J. Graham C. Merrick B. Acors S. Steel K. J. A. Hemmings O. (2020). Longitudinal evaluation and decline of antibody responses in SARS-CoV-2 infection. *medRxiv* [Preprint]. 10.1101/2020.07.09.20148429

[B83] Shoji Y. Chichester J. A. Jones M. Manceva S. D. Damon E. Mett V. (2011). Plant-based rapid production of recombinant subunit hemagglutinin vaccines targeting H1N1 and H5N1 influenza. *Human Vaccines* 7(Suppl.) 41–50. 10.4161/hv.7.0.14561 21266846

[B84] Struck A. W. Axmann M. Pfefferle S. Drosten C. Meyer B. (2011). A hexapeptide of the receptor-binding domain of SARS corona virus spike protein blocks viral entry into host cells via the human receptor ACE2. *Antiviral Res.* 94 288–296. 10.1016/j.antiviral.2011.12.012 22265858 PMC7114193

[B85] Syomin B. Ilyin Y. V. (2019). Virus-Like particles as an instrument for Vaccine production. *Mol. Biol.* 53 323–334. 10.1134/S0026893319030154 32214478 PMC7088979

[B86] Thuenemann E. C. Lenzi P. Love A. J. Taliansky M. Becares M. Zuniga S. (2013). The use of transient expression systems for the rapid production of virus-like particles in plants. *Curr. Pharm. Des.* 19 5564–5573. 10.2174/1381612811319310011 23394559

[B87] Tortorici M. A. Beltramello M. Lempp F. A. Pinto D. Dang H. V. Rosen L. E. (2020). Ultrapotent human antibodies protect against SARS-CoV-2 challenge via multiple mechanisms. *Science* 370, 950–957. 10.1126/science.abe3354 32972994 PMC7857395

[B88] Tuboly T. Yu W. Bailey A. Degrandis S. Du S. Erickson L. (2000). Immunogenicity of porcine transmissible gastroenteritis virus spike protein expressed in plants. *Vaccine* 18 2023–2028. 10.1016/S0264-410X(99)00525-3 10706964

[B89] Tusé D. Nandi S. McDonald K. A. Buyel J. F. (2020). The emergency response capacity of plant-based biopharmaceutical manufacturing – what it is and what it could be. *Front. Plant Sci.* 11:594019. 10.3389/fpls.2020.594019 33193552 PMC7606873

[B90] Vankadari N. Wilce J. A. (2020). Emerging WuHan (COVID-19) coronavirus: glycan shield and structure prediction of spike glycoprotein and its interaction with human CD26. *Emer. Microb. Infect.* 9 601–604. 10.1080/22221751.2020.1739565 32178593 PMC7103712

[B91] Walls A. C. Park Y.-J. Tortorici A. Wall A. McGuire A. T. Veesler D. (2020). Structure, function, and antigenicity of the SARS-CoV-2 spike glycoprotein. *Cell* 181 281–292. 10.1016/j.cell.2020.02.058 32155444 PMC7102599

[B92] Wang C. Zheng X. Gai W. Wong G. Wang H. Jin H. (2017). Novel chimeric virus-like particles vaccine displaying MERS-CoV receptor-binding domain induce specific humoral and cellular immune response in mice. *Antiviral Res.* 140 55–61. 10.1016/j.antiviral.2016.12.019 28040513 PMC7113847

[B93] Wang N. Shang J. Jiang S. Du L. (2020). Subunit vaccines against emerging pathogenic human coronaviruses. *Front. Microbiol.* 11:298. 10.3389/fmicb.2020.00298 32265848 PMC7105881

[B94] Webb S. R. Twyman R. M. Moloney M. (2020). Agtech infrastructure for pandemic preparedness. *Nat. Biotech.* 38 1025–1027. 10.1038/s41587-020-0654-5 32764729

[B95] Zhou T. Wang H. Luo D. Rowe T. Wang Z. Hogan R. J. (2004). An exposed domain in the severe acute respiratory syndrome coronavirus spike protein induces neutralizing antibodies. *J. Virol.* 78 7217–7226. 10.1128/JVI.78.13.7217-7226.2004 15194798 PMC421657

[B96] Zischewski J. Sack M. Fischer R. (2016). Overcoming low yields of plant-made antibodies by a protein engineering approach. *Biotechnol. J.* 11, 107–116. 10.1002/biot.201500255 26632507

